# Genome-wide analysis of AP2/ERF transcription Factors in *Cymbidium sinense* reveals their impact on orchid diversity

**DOI:** 10.3389/fpls.2025.1541308

**Published:** 2025-06-03

**Authors:** Yong-Lu Wei, Jian-Peng Jin, Jie Li, Qi Xie, Chu-Qiao Lu, Jie Gao, Gen-Fa Zhu, Feng-Xi Yang

**Affiliations:** ^1^ Guangdong Key Laboratory of Ornamental Plant Germplasm Innovation and Utilization, Environmental Horticulture Research Institute, Guangdong Academy of Agricultural Sciences, Guangzhou, China; ^2^ School of Landscape Architecture, Beijing Forestry University, Beijing, China

**Keywords:** *Cymbidium sinense*, AP2/ERF, expression pattern, floral patterning, ciselements

## Abstract

*Cymbidium sinense* is a significant traditional Chinese horticultural crop, valued both economically and ornamentally. The APETALA2/ethylene response factor (AP2/ERF) transcription factors play crucial roles in regulating growth, development, cell differentiation, and responses to both biotic and abiotic stresses in plants. However, the regulatory functions of AP2/ERF factors in *C. sinense* remain poorly understood. In the present study, 116 AP2/ERF genes were first identified from *C.sinense* genome. Based phylogenetic analysis, these genes were categorized into five groups: AP2, RAV, ERF, DREB, and Soloist. Within the ERF group, two subtypes were identified: ERF (containing six subtypes from ERF B1 to ERF B6) and DREB (containing six subtypes from DREB A1 to DREB A6), consistent with the classification in *Arabidopsis*. Significant variation was observed in gene exon-intron structures, though motifs and domain structures were highly conserved. Duplication events and collinearity analyses across five species were also conducted. Further investigations into potential cis-elements in promoter regions and expression profiles of 44 different samples, along with the analysis of 11,197 *CsAP2/ERF* target genes (functional annotation of 9,566), revealed diverse transcriptional regulatory patterns. GO enrichment and KEGG pathway analysis further elucidated these patterns. To validate transcriptome-based predictions, qRT-PCR analysis was performed on ten key *CsAP2/ERF* genes, showing high consistency with RNA-seq data. Moreover, a yeast one-hybrid (Y1H) assay confirmed that *CsAP2_51* directly binds to the promoter of *CsAG*, a key gene involved in gynostemium development, providing experimental evidence for the regulatory role of *CsAP2/ERF* in floral morphogenesis. A regulatory model was proposed to illustrate the potential roles of *CsAP2/ERF* genes in floral patterning and flower color variation. Our findings deepen the understanding of *CsAP2/ERF* gene functions in *C. sinense* and provide a valuable foundation for future studies on the molecular mechanisms underlying its growth, development, and ornamental traits.

## Introduction

The AP2/ERF (APETALA2/ethylene-responsive factor) gene family is one of the largest transcription factor families in plants, with members playing crucial roles in development, stress responses, and metabolic regulation (Xu et al., 2011; Liu et al., 2016; Hu et al., 2020; Huang et al., 2023). These genes typically contain one or two conserved AP2/ERF domains, each consisting of 60 to 70 amino acid residues. The domains adopt a characteristic three-dimensional (3D) structure formed by three β-folds and one α-helix (Allen et al., 1998). Based on the number of AP2/ERF domains and their specific functions, the AP2/ERF family is divided into five subfamilies: AP2, RAV (Related to ABI3/VP1), DREB (dehydration-responsive element-binding protein), ERF, and Soloist (Sakuma et al., 2002; Feng et al., 2020). The AP2 subfamily contains two similar AP2 domains, whereas the RAV subfamily has one AP2 domain and one B3 domain (Sakuma et al., 2002). Both the ERF and DREB subfamilies contain one AP2 domain and are further subdivided into six groups: ERF (A1–A6) and DREB (B1–B6) (Gu et al., 2017). The ERF subgroup binds to the GCC box, while the DREB subgroup interacts with DRE/CRT cis-acting elements (Hao et al., 1998; Fujimoto et al., 2000). The Soloist subfamily includes an AP2 domain but exhibits limited homology to other family members (Feng et al., 2020).

*C. sinense*, an evergreen terrestrial orchid noted for its remarkable natural diversity, winter flowering, celebrated fragrance, extended floral longevity, and moderate tolerance to shade and cold, stands out both as a premier ornamental species and as an emerging genomic model within the Orchidaceae (Hew and Wong, 2023b; Chen et al., 2024). Meanwhile, with the advent of genome sequencing, functional studies of AP2/ERF transcription factors have been conducted in a range of plant species. The first AP2/ERF gene was isolated from *Arabidopsis thaliana*, where it was found to regulate flower development (Jofuku et al., 1994; Elliott et al., 1996). This was followed by the identification of DREB proteins involved in drought and cold stress responses (Liu et al., 1998; Sakuma et al., 2002). In tobacco, four ERF proteins were found to regulate ethylene-responsive genes (Ohme-Takagi and Shinshi, 1995), and the roles of ERF1 and ERF6 in regulating defense responses, growth, and development were characterized (Berrocal-Lobo et al., 2002; Lorenzo et al., 2004; Cheng et al., 2013). Additional genome-wide analyses have shed light on the functional diversity and evolutionary history of the AP2/ERF gene family. For example, in tomato, AP2/ERF genes regulate processes such as carotenoid biosynthesis, fruit ripening, and stress responses (Liu et al., 2014; Chen et al., 2023a), as well as salt tolerance (Bouzroud et al., 2018; Jiang et al., 2024), and influence hypocotyl elongation and plant height (Chen et al., 2023b). In apples, AP2/ERF proteins modulate carotenoid accumulation, affecting fruit color (Dang et al., 2021; Ampomah-Dwamena et al., 2022). In maize, the *BRANCHED SILKLESS1* (*BD1*)/*FRIZZY PANICLE* (*FZP*) gene, a member of the AP2/ERF family, is essential for promoting determinacy and producing sex organs (Du et al., 2022). However, little is known about how the AP2/ERF gene family regulates development in *C. sinense*. Considering the AP2/ERF superfamily plays a central role in floral organ patterning, pigment biosynthesis, and abiotic stress responses, all of which are key targets for orchid improvement, a comprehensive identification and functional annotation of *AP2*/*ERF* genes in *C. sinense* has become an urgent priority. The function of AP2/ERF genes in *C. sinense* makes it an ideal candidate for functional genomics and molecular breeding, offering potential for the development of new cultivars with enhanced ornamental features, improved environmental resilience, and optimized flowering schedules.

In this study, we investigate the AP2/ERF transcription factors in *C. sinense* by analyzing both transcriptomic and genomic data. We identified 116 AP2/ERF genes from the *C. sinense* genome and studied their gene structure, chromosomal localization, conserved motifs, phylogenetic relationships, duplication events, and cis-acting elements. Additionally, transcriptome data were used to analyze the expression patterns of these genes during various stages of development. Finally, we propose a model of the potential regulatory roles of *CsAP2/ERF* genes in the floral variations observed in *C. sinense*. This research not only enhances our understanding of the roles of *CsAP2/ERF* genes but also provides valuable resources for the genetic improvement of orchids.

## Materials and methods

### Plant materials, growth conditions, and treatment

In this study, Orchid plants (C. sinensis ‘Qihei’) were grown in a growth chamber at the Institute of Environmental Horticulture, Guangdong Academy of Agricultural Sciences (Guangzhou, China). The chamber was maintained at 80% relative humidity with a temperature regime of 25°C during a 16-hour light cycle and 18°C during an 8-hour dark period. Adult plants, aged two years, were used for sample collection. We harvested roots, stems, leaves, flowers, and fruits, which were then rapidly frozen in liquid nitrogen and stored at -80°C for further analysis. To investigate the effects of stress treatments, *C. sinense* plants were sprayed weekly with a 100 μM solution of abscisic acid (ABA) for one month. After the treatment period, leaves and flowers were collected, frozen in liquid nitrogen, and stored at -80°C. Untreated plants were used as controls. For RNA-seq analysis, both ABA-treated and control groups included three independent biological replicates to ensure statistical robustness.

### Identification of AP2/ERF genes from *C.sinense*

The genome sequences of *C. sinense* were obtained from our laboratory (Yang et al., 2021). To identify AP2/ERF genes, protein sequences of AP2/ERF family members from *Oryza sativa* and *Arabidopsis thaliana* were retrieved from the database (https://www.ncbi.nlm.nih.gov/). These sequences were used as queries for BLASTP searches against the *C. sinense* genome with an E-value threshold of 1e-5. In parallel, the Hidden Markov Model (HMM) profile of the AP2 domains (PF00847) were obtained from the PFAM database (https://pfam.xfam.org/), and used to perform HMMER-based domain searches. Candidate proteins identified by both methods were further examined to confirm the presence of conserved AP2 domains using the Conserved Domains Database (CDD) online tool (https://www.ncbi.nlm.nih.gov/cdd/). A total of 116 *CsAP2/ERF* genes were identified. The molecular weights and isoelectric points of these proteins were predicted using the ProtParam tool on the ExPASy proteomics server (https://web.expasy.org/compute_pi/). Subcellular localization was predicted using the CELLO tool (http://cello.life.nctu.edu.tw/).

### Phylogenetic analysis of *C.sinense* AP2/ERF genes

To explore the phylogenetic relationships of the *CsAP2/ERF* genes, we performed multiple sequence alignments using ClustalW (Larkin et al., 2007) with default parameter. Phylogenetic trees were constructed using the IQ-TREE v.1.6.9 (Nguyen et al., 2015) under a VT+F+I+G4 model, which was selected as the best-fit substitution model based on ModelFinder (Kalyaanamoorthy et al., 2017). The tree was generated with 1000 ultrafast bootstrap replicates following multiple sequence alignment using pairwise deletion.

### Gene structure and conserved motif analyses

Conserved motifs in *CsAP2/ERF* proteins were identified using the MEME Suite v.5.0.5 (Bailey et al., 2009). The following parameters were applied: (1) zero or one occurrence of each motif per sequence; (2) a maximum of 25 motifs; (3) motif width ranging from 6 to 50 amino acids; and (4) E-value < 0.05. Gene structures were visualized using the GSDS 2.0 tool (http://gsds.cbi.pku.edu.cn/). Phylogenetic trees, conserved motifs, and gene structures were integrated using TBtools v1.09876 software (Chen et al., 2020). The 3D structures of the *CsAP2/ERF* proteins were modeled using AlphaFold2 (Cramer, 2021) and visualized with PyMOL (Janson et al., 2016).

### Chromosomal distribution, duplication, and collinearity analysis of AP2/ERF superfamily genes

Chromosomal distribution of the identified *CsAP2/ERF* genes was mapped against the reference *C. sinense* genome. Gene duplication events were analyzed using MCScanX (Wang et al., 2012), and syntenic relationships between *CsAP2/ERF* genes and those from selected plant species were identified. Tandem duplications were defined as pairs of genes with greater than 40% similarity and separated by four or fewer loci, while segmental duplications were those separated by more than five genes. The results were visualized using Circos software (version 0.69-9) (Krzywinski et al., 2009).

### Transcriptome-based expression profiling and quantitative real-time PCR validation of CsAP2 genes

Transcriptomic datasets of *C. sinense* across various tissues (root, stem, leaf, flower, and fruit), floral developmental stages, floral color and morphological variants, and dissected floral organs (sepal, petal, labellum, and gynostemium) were obtained from NCBI BioProject PRJNA743748. Raw reads were preprocessed using Trimmomatic v0.39 to remove adapter sequences and low-quality bases (Bolger et al., 2014). Clean reads were subsequently aligned to the *C. sinense* reference genome using HISAT2 (Kim et al., 2019). Gene expression levels were quantified and normalized to FPKM (Fragments Per Kilobase of transcript per Million mapped reads) using RSEM v1.3.0 (Li and Dewey, 2011). For visualization, expression values were transformed using log_2_(FPKM + 1) and displayed as heatmaps generated with the R package the R package pheatmap (Kolde, 2015).

To validate RNA-seq-based expression profiles, five key *CsAP2* genes were selected for qRT-PCR analysis. Total RNA was extracted from representative tissue samples, and cDNA synthesis was performed using standard protocols. Gene-specific primers used for qRT-PCR are listed in **Supplementary Table S1**.

### Identification and characterization of target genes of *CsAP2/ERF*

To identify potential target genes regulated by *CsAP2/ERF* factors, we analyzed the 2000-bp upstream regions of transcription start sites (considered putative promoter regions). AP2/ERF binding site motifs, such as DRE/CRT (G/ACCGAC, MA1670.1 and MA1670.2) and GCC-Box (AGCCGCC, MA0567.1, MA1049.1, and MA1049.2), were retrieved from the JASPAR CORE database (https://jaspar.genereg.net/) (Khan et al., 2018). The FIMO tool (part of the MEME Suite) was used to identify AP2-binding motifs within the *C. sinense* promoter regions, with a significance threshold of p < 1 × e-4. We further predicted the potential regulatory effects of representative *CsAP2/ERF* proteins on the identified target genes using AlphaFold3 (Abramson et al., 2024). Candidate genes were functionally annotated using Gene Ontology (GO) and Kyoto Encyclopedia of Genes and Genomes (KEGG) databases.

### Yeast one-hybrid assay

The coding sequence (CDS) of *CsAP2_51* was cloned into the pB42AD vector. Promoter fragments of *CsAG* (*Mol017710*) were amplified from *C. sinense* genomic DNA and inserted into the pLacZi vector. Yeast strain EGY48 was co-transformed with combinations of these plasmids or empty vector controls using the Yeastmaker™ Yeast Transformation System 2 (Clontech, USA). Transformants were grown initially on SD/-Trp/-Ura medium at 30°C for three days, then transferred onto SD/-Trp/-Ura/Gal/Raf medium containing X-Gal for another three days. Blue colony formation indicated positive DNA-protein interactions.

## Results

### Basic characterization of AP2 genes in 
*C. sinense*

In this study, candidate genes from the AP2/ERF superfamily were initially identified using the *Cymbidium sinense* genomic database. To confirm the presence of the AP2/ERF domain, all protein sequences of the putative *CsAP2/ERF* genes were analyzed using the SMART search tool and the NCBI Conserved Domain Database (CDD). A total of 116 *CsAP2/ERF* genes were identified, each encoding proteins with one or more AP2/ERF domains. Based on the number of AP2/ERF domains and the similarity of amino acid sequences, these 116 *CsAP2/ERF* proteins were classified into five distinct families: AP2 (13 genes), ERF (65 genes), DREB (34 genes), RAV (2 genes), and Soloist (2 genes). Among the AP2 family members, 6 out of 13 contained two AP2/ERF domains. The RAV family included 2 genes, each with both an AP2/ERF and a B3 domain. The Soloist family contained 2 genes, both of which featured a single AP2/ERF domain and showed the highest homology to the *Arabidopsis thaliana* gene At4g13040 (Rao et al., 2015) (**Table 1**). As shown in **Supplementary Table S2**, the proteins exhibited a wide range of characteristics, with sequence lengths varying from 99 to 1,128 amino acids and molecular weights ranging from 11.32 kDa to 128.12 kDa.

**Table 1:** Summary of the AP2/ERF superfamily in Cymbidium sinense genome.

Classification	Group	No.
AP2	Double AP2/ERF domain	6
	Single AP2/ERF domain	7
DREB	A1	4
	A2	5
	A3	1
	A4	9
	A5	8
	A6	7
ERF	B1	9
	B2	6
	B3	17
	B4	14
	B5	10
	B6	9
RAV		2
Soloist		2
	Total	116

### Phylogenetic analysis of *C. sinense* AP2/ERF proteins

**Figure 1:**
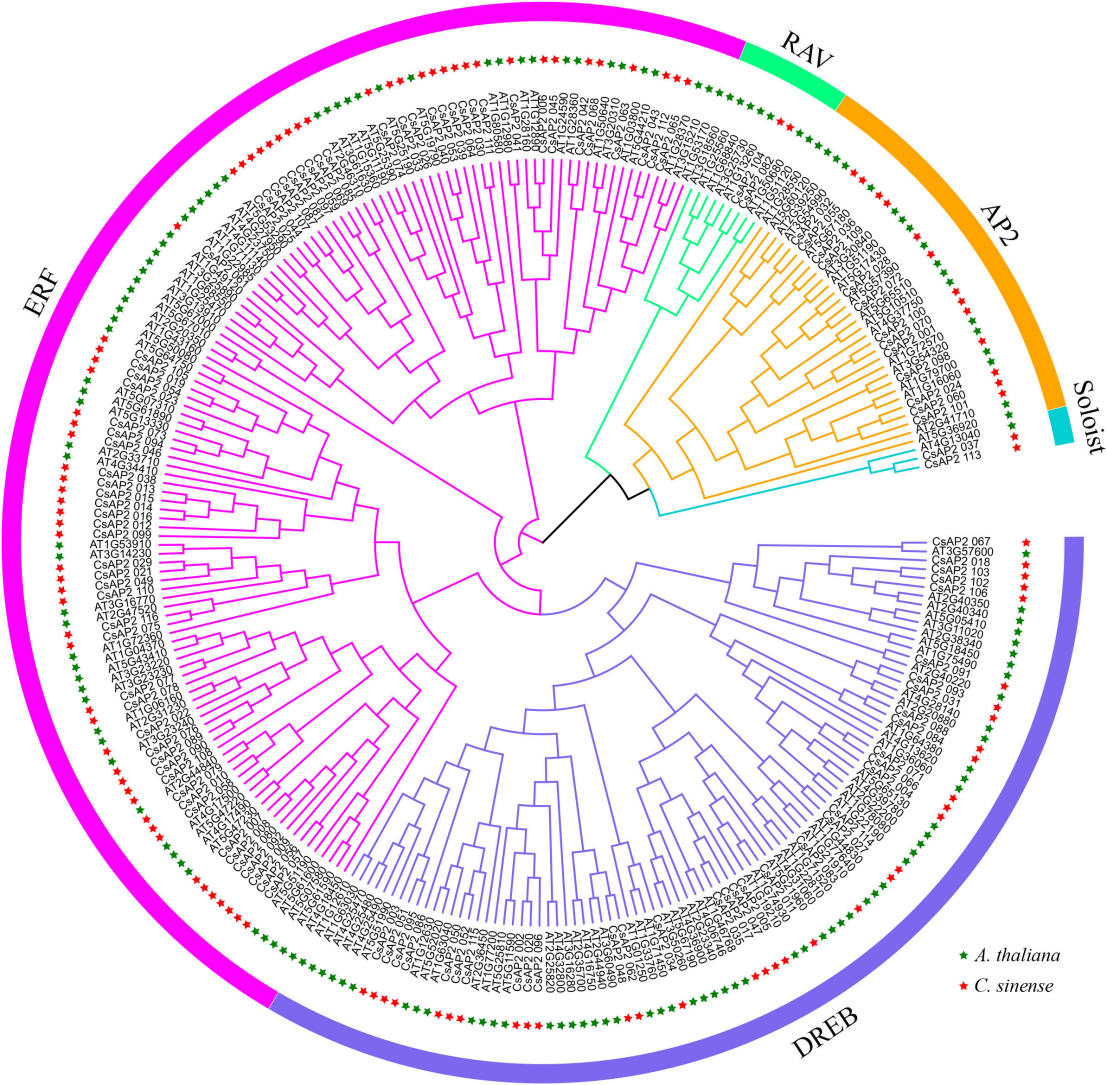
Phylogenetic tree of *AP2/ERF* genes in *C. sinense* and *A. thaliana*. The ERF, DREB, AP2, RAV, and Soloist families are represented in different colors.

A phylogenetic tree was constructed using the protein sequences of AP2/ERF genes from *C. sinense* and *Arabidopsis thaliana*. The analysis revealed that the *CsAP2/ERF* proteins clustered into distinct clades, including ERF, DREB, AP2, RAV, and Soloist (**Figure 1**). Notably, the majority of the *CsAP2/ERF* proteins were classified into the ERF and DREB families, a pattern consistent with previous studies (Nakano et al., 2006; Cao et al., 2020).

### Domains and gene structure analysis

To investigate the diversity and similarities within the AP2/ERF transcription factors in *C. sinense*, we analyzed the domain organization and exon-intron structure of the *CsAP2/ERF* genes based on their phylogenetic distribution (**Supplementary Figure 1**). The structural domains of the *CsAP2/ERF* proteins revealed two main conserved regions: (1) a 60–70 amino acid long AP2 region (motifs 1, 2, 3, and 4) located in the N-terminal region, which serves as the DNA-binding domain, and (2) a 100–120 amino acid long B3 region (motif 25), a distinctive feature of the RAV subfamily (**Supplementary Table S3**).

Exon-intron structure analysis showed significant variation among the *CsAP2/ERF* genes in *C. sinense*. A total of 74 genes were intronless, while the remaining 42 genes contained between 1 and 11 introns. The number of exons ranging from 1 to 12 (**Supplementary Figure 1**). Notably, all AP2 subfamily members contained four or more exons, suggesting that the exon distribution within the AP2 subfamily is highly conserved. In contrast, most members of the other subfamilies had a single exon containing the AP2 domain in the exon region (**Supplementary Figure 1**). This variation in exon-intron structure did not seem to affect the conservation of the key exons in the AP2 subfamily. Interestingly, proteins with similar amino acid sequences were classified into the same subfamily and likely share similar functions. However, proteins with the same domain may exhibit distinct functions due to conformational differences.

**Figure 2:**
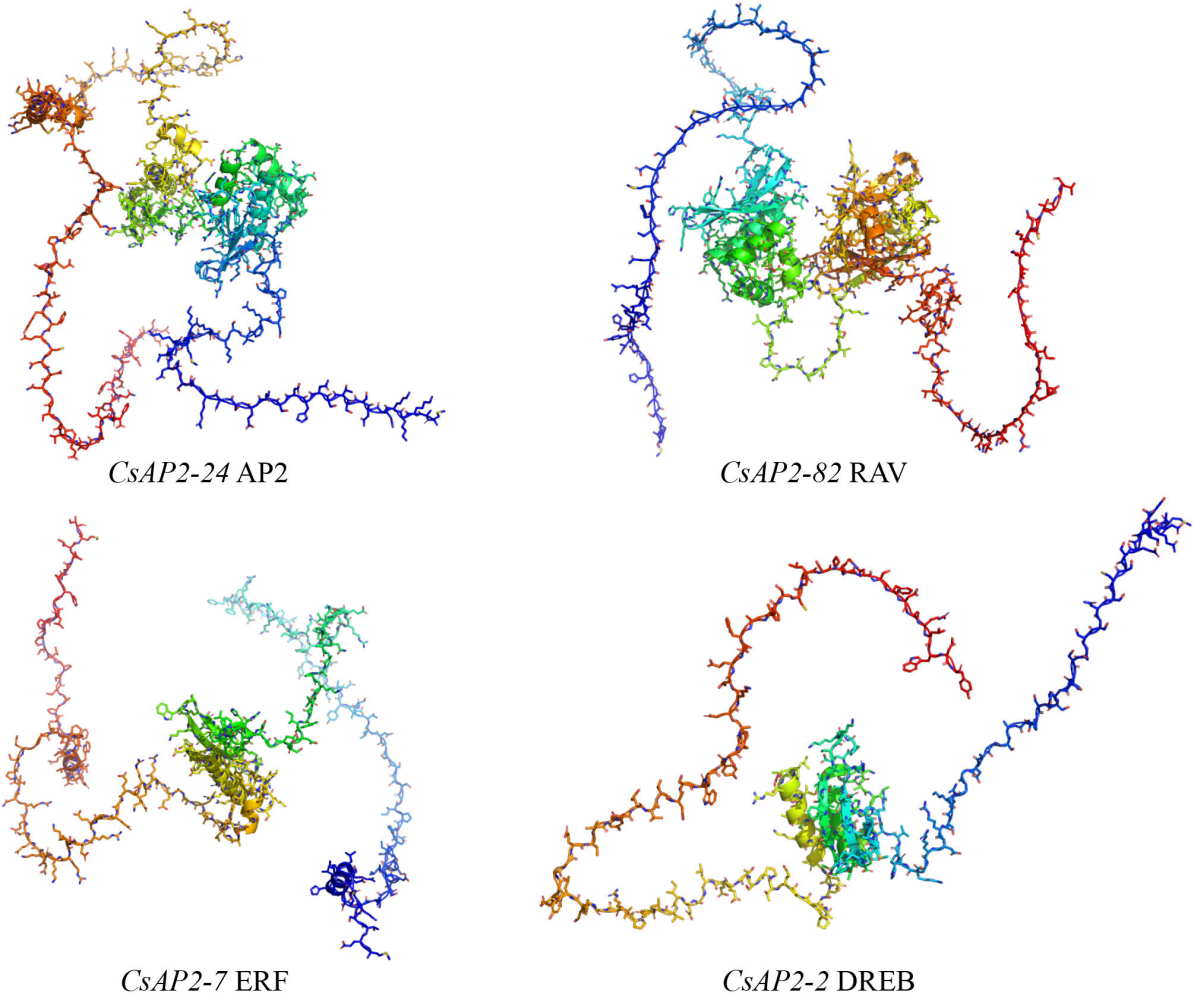
Structural predictions of CsAP2/ERF proteins. The color gradient from red to blue represents the orientation from the N-terminal to the C-terminal of each protein.

To further explore the structure-function relationship, we selected four representative *CsAP2/ERF* proteins from the five subfamilies for 3D structure analysis. The predicted structures were modeled using AlphaFold2 (**Figure 2**). The 3D structure analysis revealed that all proteins contained a highly conserved AP2/ERF domain, typically arranged in a parallel α-helix and three anti-parallel β-sheets. Further inspection of the AP2/ERF domain highlighted two key regions: the YRG region (located in the N-terminal) and the RAYD region (near the C-terminal). The YRG region, approximately 20 amino acids in length, is rich in hydrophilic and basic amino acids, while the RAYD region, spanning 40 amino acids, is involved in protein-protein interactions through an α-helix structure. Additionally, the AP2 subfamily members were characterized by two AP2/ERF domains connected by a 25-amino acid linker, which is responsible for organizing the DNA-binding domain (Klucher et al., 1996). These findings underscore the structural consistency and provide essential insights into the molecular functions of *CsAP2/ERF* proteins.

### Chromosomal locations, duplication, and collinearity of the AP2/ERF TFs in *C. sinense*

**Figure 3:**
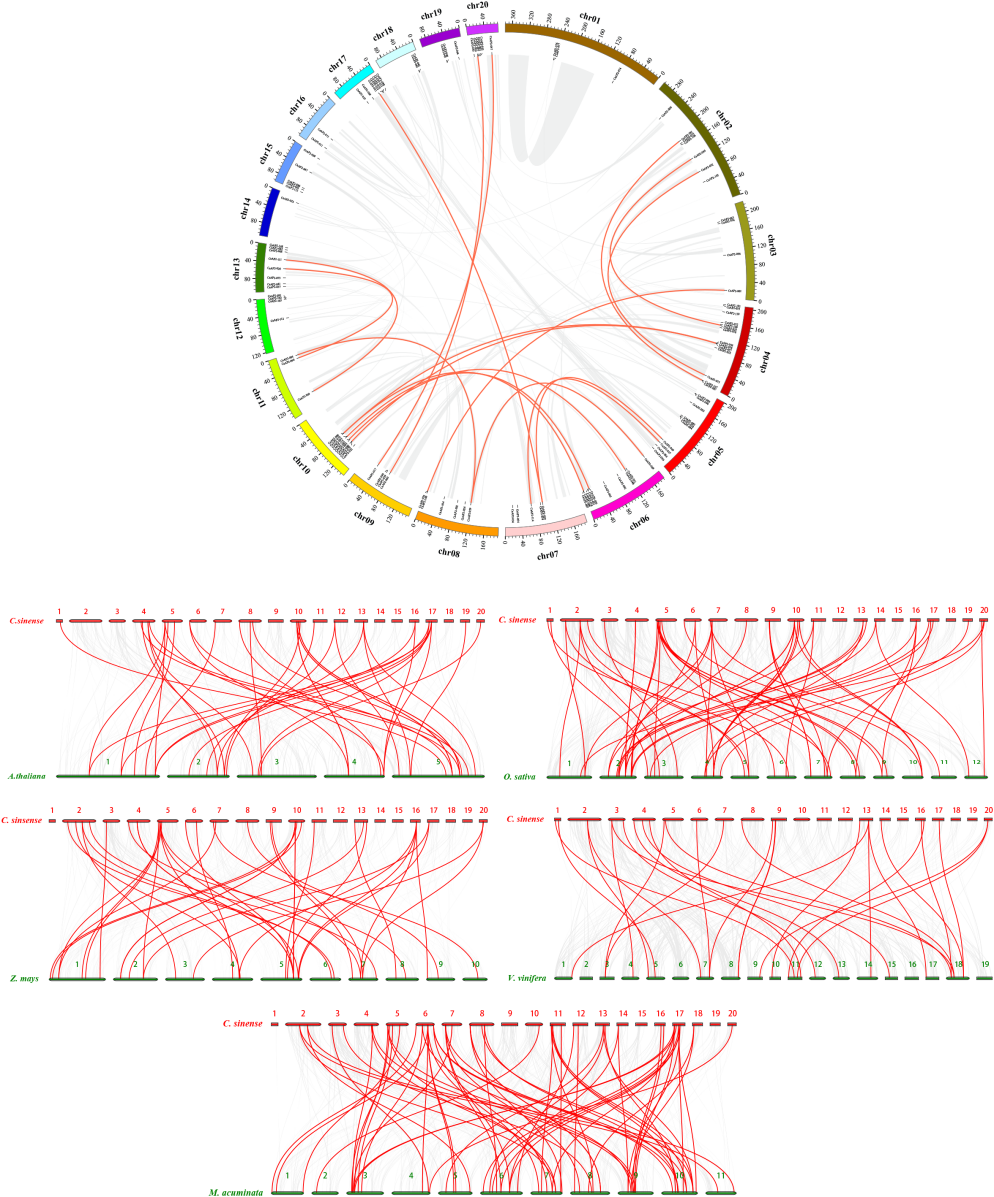
Chromosomal distribution and gene duplication of AP2 genes in *C. sinense*, and synteny of the *CsAP2/ERF* genes with those of *A. thaliana*, *O. sativa*, *Z. mays*, *V. vinifera*, and *M. nana*. The scale is in megabases (Mb), with chromosome lengths indicated at the top. Gene positions are shown with gray lines, and paralogous *CsAP2* genes are connected by red lines.

The 116 AP2/ERF transcription factor (TF) genes in *C. sinense* are distributed across the twenty chromosomes, with their physical locations displayed in **Figure 3**. The number of AP2/ERF genes on each chromosome ranges from 1 to 14. Chromosomes 4, 5, and 6 harbor the highest number of AP2/ERF genes, containing 14, 10, and 10 genes, respectively. Among all chromosomes, chromosomes 14 and 16 contain the lowest number of AP2/ERF genes, with 11 and 10 genes, respectively. Notably, the distribution of AP2/ERF genes is not random, as several gene clusters referred to as “hot regions”—are present on specific chromosomes. For example, chromosome 10 contains 9 AP2/ERF genes within a small region (~41.13 Mb), and similar gene clusters are also found on chromosomes 5 and 6 (**Figure 3**).

To investigate gene duplication, we identified 18 pairs of duplicated genes that contributed to the expansion of the *CsAP2/ERF* gene family. These duplications are spread across different chromosomes and are primarily the result of segmental duplications. For instance, *CsAP2* genes 59, 69, 81, 85, and 87 from the ERF-B5 group are located on separate chromosomes: *CsAP2_59* on chromosome 8, *CsAP2_69* on chromosome 6, *CsAP2_81* on chromosome 2, *CsAP2_85* on chromosome 11, and *CsAP2_87* on chromosome 4. These genes represent products of segmental duplications across the genome.

**Figure 4:**
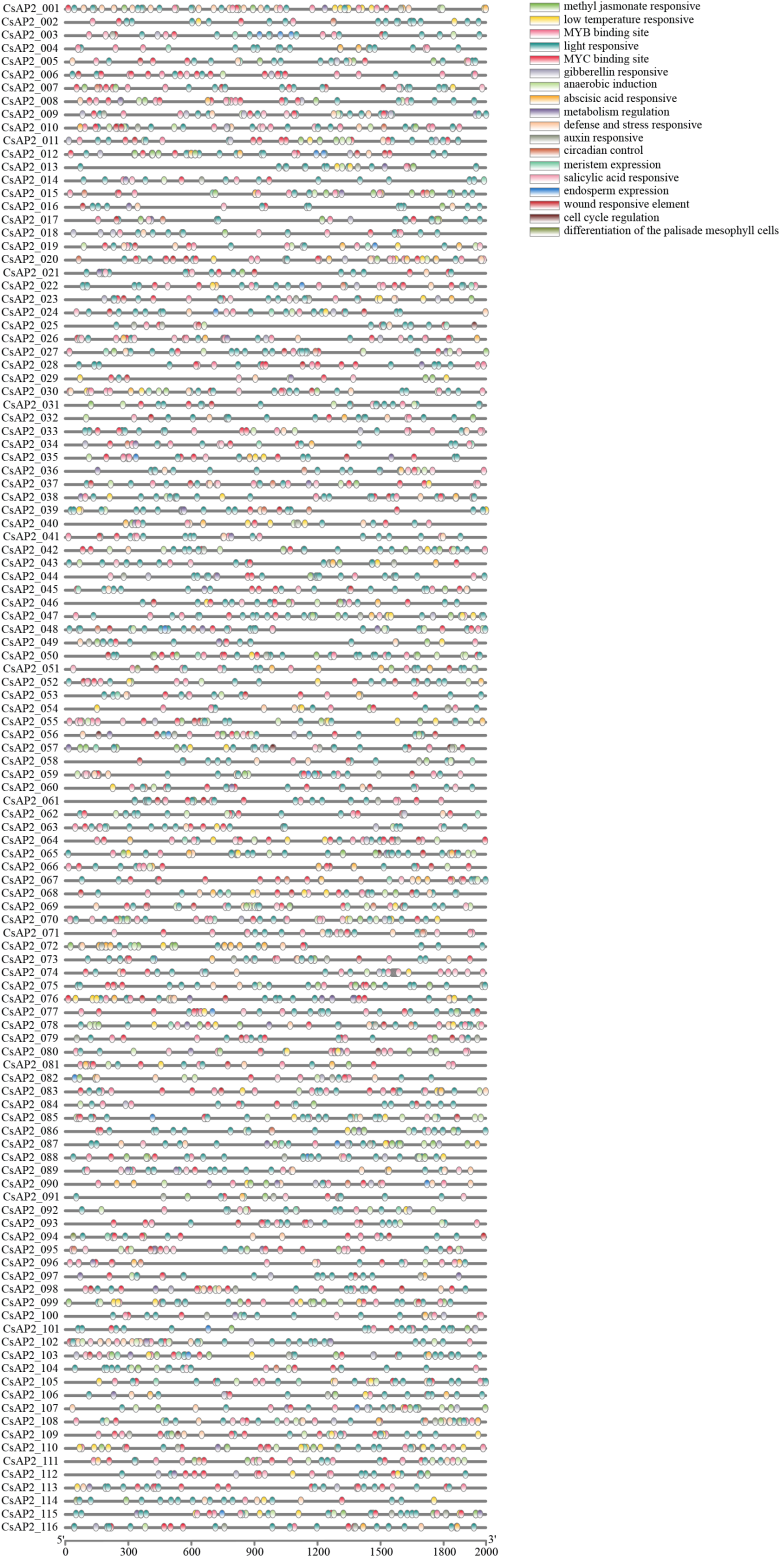
Cis-acting elements analysis of *CsAP2/ERF* promoters. The 2,000 bp upstream sequence from the transcription initiation site of CsAP2/ERF genes was analyzed.

We also explored the orthologous relationships between *C. sinense* and several other plant species, including dicotyledons (*Arabidopsis thaliana* and *Vitis vinifera*) and monocotyledons (*Oryza sativa*, *Musa nana*, and *Zea mays*), to better understand the evolutionary dynamics of the AP2/ERF gene family (**Figure 3**). A total of 235 *CsAP2/ERF* genes exhibited syntenic relationships with genes from *A. thaliana* (40 genes), *V. vinifera* (29 genes), *O. sativa* (57 genes), *M. nana* (68 genes), and *Z. mays* (41 genes). Notably, three *CsAP2/ERF* genes (*CsAP2_66*, *CsAP2_71*, and *CsAP2_73*) showed collinearity with all five species (**Supplementary Table S4**). As shown in **Figure 4**, the number of orthologous gene pairs between *C. sinense* and monocot species is significantly higher than those with dicot species. Some collinear genes were found exclusively between *C. sinense* and other monocotyledons.

### Expression profiles of *CsAP2/ERF* genes in *C. sinense*

**Figure 5:**
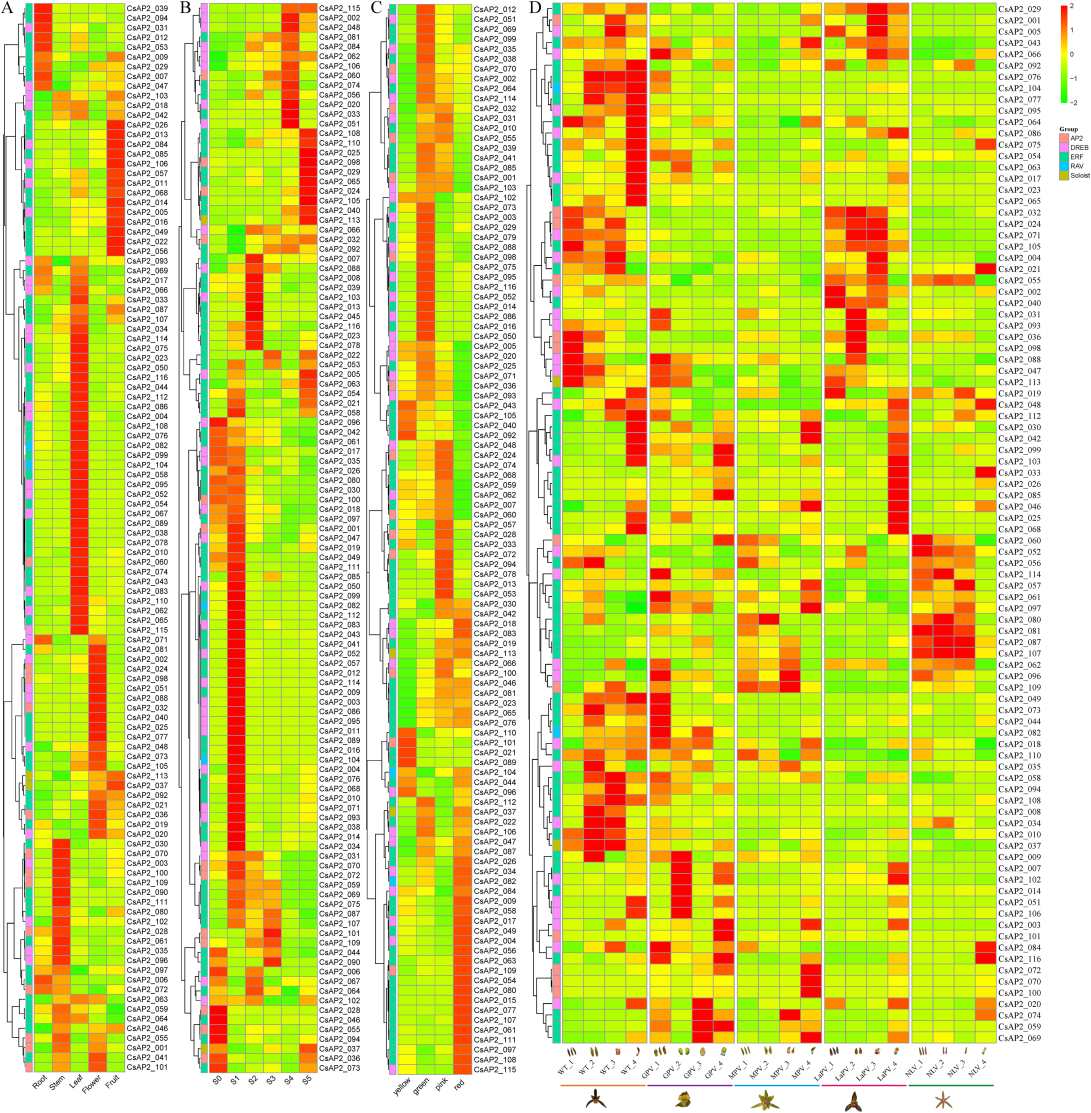
Expression profiles of the *CsAP2/ERF* genes in *C. sinense*. **(A)** Hierarchical clustering of the expression profiles in five major tissues: root, stem, leaf, flower, and fruit tissues. **(B)** Expression patterns across six stages of floral development: S0 (dormant lateral buds), S1 (1–5 mm floral buds), S2 (6–10 mm), S3 (11–15 mm), S4 (16–20 mm), and S5 (blooming flowers). **(C)** Expression profiles among different flower colors: yellow, green, pink, and red. **(D)** Expression patterns in different floral organs from four floral morphotypes: LaPV (labellum-like perianth variety), MPV (multi-perianth variety), NLV (null-lip variety), and GPV (gynostemium-like perianth variety). For **(D)** numerals 1 to 4 represent sepal, petal, labellum, and gynostemium, respectively. Expression levels are shown as log_2_(FPKM + 1).

The expression profiles of *CsAP2/ERF* genes in various tissues of *C. sinense*, including roots, stems, leaves, flowers, and fruits, were investigated based on previous transcriptomic data (**Figure 5**). The results revealed substantial variation in the expression levels of the 116 identified *CsAP2/ERF* genes (**Supplementary Table S5**). Among them, 110 genes were expressed in at least one of the five tissue types, while six genes were not detected in any tissue. A total of 70 genes were expressed across all tested tissues, although some of them exhibited relatively low expression levels.

To further analyze the transcriptional profiles of *CsAP2* genes, we clustered their expression patterns across all tissues (**Figure 5**). Several genes showed tissue-specific expression or high expression levels in particular tissues. For example, five genes (*CsAP2_12*, *CsAP2_31*, *CsAP2_39*, *CsAP2_53*, *CsAP2_94*) were predominantly expressed in roots. Eleven genes (e.g., *CsAP2_03*, *CsAP2_61*) showed high expression levels in stems compared to other tissues. Furthermore, 33 genes (e.g., *CsAP2_04*, *CsAP2_99*) were preferentially expressed in leaves, suggesting their potential involvement in leaf development. Additionally, 10 genes (e.g., *CsAP2_02*, *CsAP2_24*) showed predominant expression during flower development, while another 10 genes (e.g., *CsAP2_05*, *CsAP2_85*) were mainly expressed during fruit development. The full list of tissue-specific genes is available in **Supplementary Table S6**.

In flowers, further analysis was performed to investigate expression patterns across various floral tissues (sepals, petals, lips, and gynostemiums) and stages of flower development (dormant lateral buds, 1–5 mm, 6–8 mm, 11–15 mm, 16–20 mm floral buds, and blooming flowers). The expression of *CsAP2/ERF* genes was also analyzed based on flower color (yellow, green, pink, red) and flower patterning (standard type, gynostemium-like perianth variety, multi-perianth variety, labellum-like perianth variety, and null-lip variety). Genes were grouped into clusters based on their expression patterns (**Figure 5**), with most showing higher expression in the 1–5 mm floral bud stage compared to other stages of flower development. Fewer genes were detected in yellow flowers compared to other flower colors. Interestingly, tissue-specific expression was observed in various floral tissues among different flower varieties. For example, *CsAP2_56* and *CsAP2_51* exhibited higher expression in the petal and gynostemium, respectively, suggesting their potential roles in floral tissue differentiation.

Further analysis was conducted to characterize the expression patterns of *CsAP2* genes across distinct leaf color variants (green, yellow, and red leaves) in *C. sinense*. Genes were grouped based on their specific expression profiles (**Supplementary Figure 2**). Notably, six *CsAP2* genes exhibited higher expression specifically in yellow leaves, while another distinct set of six genes showed elevated expression exclusively in red leaves. In contrast, only one gene, *CsAP2_61*, demonstrated high expression uniquely in green leaves. These results suggest that different subsets of *CsAP2* genes potentially regulate leaf color differentiation in *C. sinense*.

To validate the reliability of the transcriptome data, five *CsAP2* genes showing differential expression across various tissues and phenotypic variants were selected for qRT-PCR analysis. The qRT-PCR results were generally consistent with the expression patterns observed in the transcriptome dataset (**Supplementary Figure 3**), thereby confirming the accuracy of the RNA-seq data. These findings support the selection of these candidate *CsAP2* genes for further investigation into their roles in the growth, development, and natural variation of *C. sinense*.

To further explore the potential regulatory roles of *CsAP2/ERF* genes, we examined their expression patterns under ABA treatment, a key phytohormone involved in flowering regulation and abiotic stress responses in *C. sinense*. The expression profiles revealed pronounced transcriptional changes in response to ABA. For instance, *CsAP2_3* exhibited more than a 700-fold decrease in expression in flowers without treatment, while *CsAP2_52* showed a 10-fold increase in leaves. After ABA treatment, *CsAP2_74* demonstrated a more than 3000-fold increase in expression in leaves, and *CsAP2_108* showed over a 77-fold increase in flowers. These results suggest that *CsAP2/ERF* genes are likely involved in the regulation of floral and leaf development in *C. sinense*, particularly through ABA-responsive pathways (**Supplementary Figure 4**, **Supplementary Table S5**).

### Cis-acting elements analysis of CsAP2 gene family

Plants regulate gene expression through two key mechanisms: cis-acting elements and trans-acting elements (Stamatoyannopoulos, 2010). These mechanisms interact to modulate gene expression, either enhancing or repressing it. Cis-acting elements, found in both coding and non-coding regions of genes, particularly the promoter regions, are involved in processes such as stress response, tissue-specific expression, and environmental adaptability.

To gain insights into the regulatory roles of CsAP2/ERF genes, we analyzed the 2000-bp upstream sequence from the start codon (ATG) of each gene using the PlantCARE database. Our analysis revealed that a diverse range of cis-acting elements are present in the promoter regions of *CsAP2*/*ERF* genes (**Figure 4**). These elements can be grouped into major categories: regulatory elements related to hormone responses, such as methyl jasmonate (597), abscisic acid (327), and gibberellin (88); regulatory elements related to stress responses, such as low temperature (127), defense and stress (167), and wound responses (103); regulatory elements serving as transcription factor binding sites, such as MYB (536) and MYC (410); and regulatory elements related to growth and developmental processes, such as light-responsive (1328), metabolism regulation (80), and meristem expression (49) (**Figure 4** and **Supplementary Table S7**). Significantly, hormone-responsive elements suggest roles for *CsAP2/ERF* genes in hormonal pathways, while stress-responsive elements indicate their involvement in adaptation to biotic and abiotic stress. Additionally, elements related to growth and developmental processes emphasize their broader regulatory roles during plant development. Collectively, these findings underscore the multifaceted regulatory potential of *CsAP2/ERF* genes across diverse biological contexts.

### Uncovering and characterizing target genes of CsAP2/ERF in *Cymbidium*

**Figure 6:**
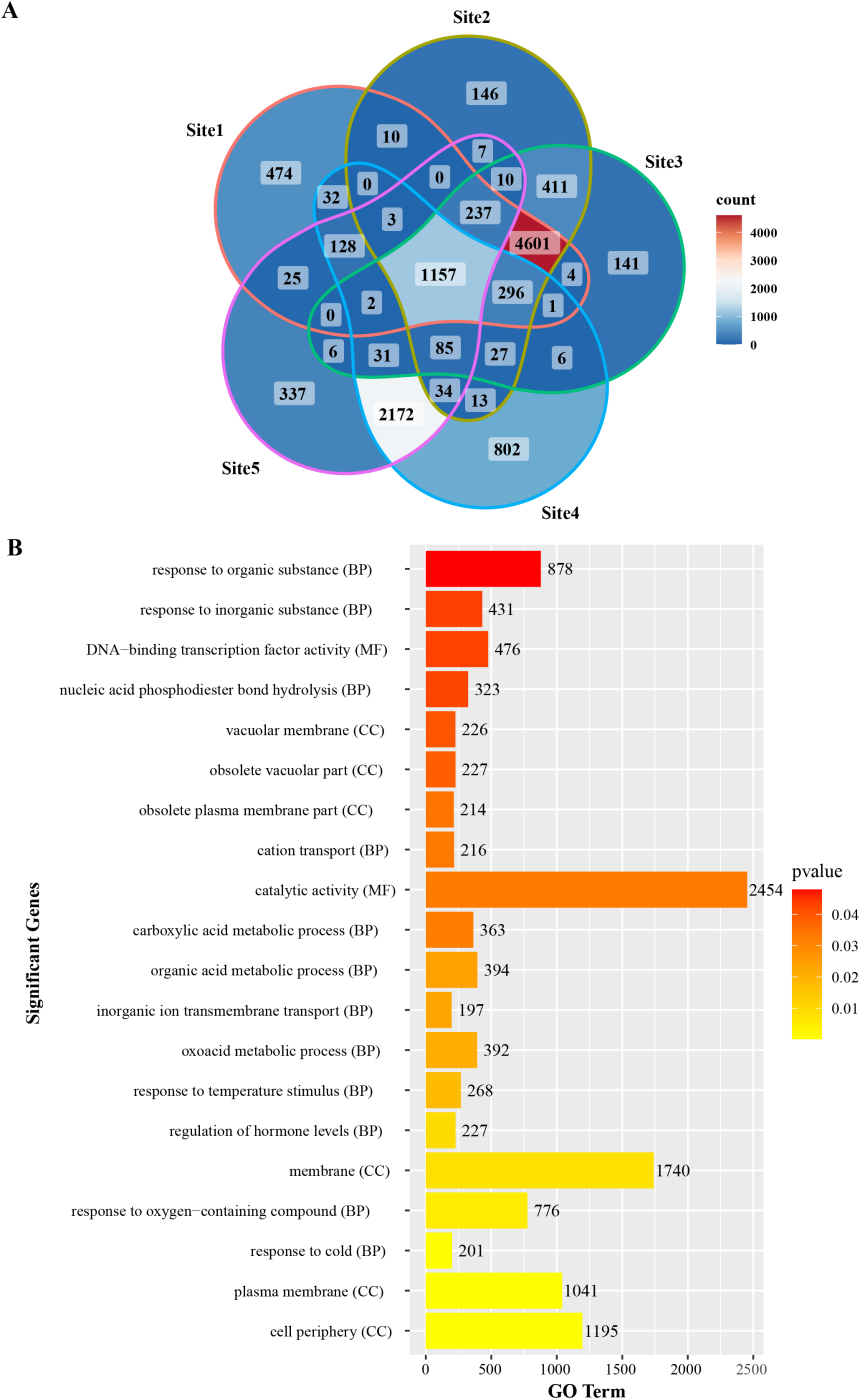
**(A)** Venn diagram of consensus motifs in different *AP2/ERF* DNA binding sites (Site 1, MA0567.1; Site 2, MA1049.1; Site 3, MA1049.2; Site 4, MA1670.1; Site 5, MA1670.2) in *C. sinense*. **(B)** Top 20 enriched Gene Ontology (GO) terms for candidate CsAP2/ERF target genes. MF, molecular function; CC, cellular component; BP, biological process.

To investigate potential downstream genes regulated by *CsAP2/ERF* transcription factors in *C. sinense*, we analyzed the 2000-bp upstream promoter sequences of *C. sinense* genes using the JASPAR database to identify consensus AP2/ERF binding motifs. This analysis revealed a total of 11,197 potential target genes, which are illustrated in **Figure 6A** and listed in **Supplementary Table S8**. We further explored the biological roles of these target genes through Gene Ontology (GO) enrichment and Kyoto Encyclopedia of Genes and Genomes (KEGG) pathway analyses. The GO analysis, which assigned functional annotations to 5420 target genes (**Supplementary Table S9**), showed a broad range of protein functions. The top 20 enriched GO terms are presented in **Figure 6B**, providing insight into the functional diversity of the target genes. In addition to GO analysis, we performed KEGG pathway enrichment analysis, which identified 3075 target genes significantly associated with various biological pathways (**Supplementary Table S10**). The most notable pathways include: metabolism (1485) and Ribosome biogenesis (173) with statistical significance (p < 0.05). Both GO and KEGG analyses suggest that CsAP2/ERF genes may play crucial roles in regulating metabolic processes, ribosome biogenesis, and other vital pathways.

Furthermore, we found that these target genes are associated with over 1,000 distinct protein domains, such as protein kinase, MADS-box, cytochrome P450, and zinc finger domains. The diversity of these domains indicates that CsAP2/ERF transcription factors could regulate a wide range of target genes, impacting multiple aspects of *C. sinense* growth and development (**Supplementary Table S11**).

To provide empirical support for the bioinformatic predictions of regulatory interactions, we selected *CsAG* (*Mol016808*), a key gene controlling floral development that contains predicted AP2 transcription factor binding motifs within its promoter region. Integration of transcriptomic analyses with RT-qPCR validation confirmed tissue-specific expression patterns of both *CsAP2_51* and *CsAG* (**Supplementary Figure 5**). In yeast one-hybrid assays, yeast cells co-transformed with the pB42AD-*CsAP2_51* activation construct and the pLacZi reporter harboring the *CsAG* promoter fragment showed robust growth on selective medium and developed distinct blue colonies on X-Gal-containing medium. This result conclusively demonstrates that *CsAP2_51* can transcriptionally activate the promoter of *CsAG*. These findings experimentally validate the predicted AP2 regulatory module and substantiate the functional relevance of candidate gene networks identified through integrative genomic approaches in this study.

## Discussion

### CsAP2 gene family structure and characteristics

The AP2/ERF superfamily among the largest families of transcription factors (TFs) plants and orchestrates diverse developmental processes (Licausi et al., 2013; Feng et al., 2020). While most studies have focused on model and crop plants, genome-wide identification of AP2/ERF genes in ornamental plants remains limited. In this study, we identified 116 *CsAP2/ERF* genes and characterized their structures, revealing common features across the family. Notably, RAV TFs, characterized by a B3 domain in their C-terminus, are known to be involved in defense responses against bacterial and fungal infections (Xie et al., 2024). Interestingly, *CsRAV* contains two AP2 domains, unlike other species that typically have only one, which may suggest it has a broader functional capacity. Structural analysis also revealed that 74 *CsAP2* genes lacked introns, accounting for 63.73% of the family members. The RAV subfamily was also intronless, while the AP2 subfamily contained more than three introns, a pattern similar to that observed in *Rhododendron* (Guo et al., 2023). These structural signatures provide a framework for understanding the lineage-specific expansion and regulatory innovation of this gene family in orchids.

To further explore the structural features of the CsAP2/ERF proteins, we modeled their tertiary conformations using AlphaFold2. The predicted structures confirmed the presence of conserved AP2 DNA-binding domains across the family. In addition, we observed that many CsAP2/ERF proteins possess extended unstructured C-terminal regions enriched with negatively charged residues. These disordered regions resemble structural features reported in *A. thaliana*, where they are known to enhance the efficiency of DNA target searching by increasing binding flexibility (Wang et al., 2023). Such intrinsically disordered tails can also contribute to protein stability, mediate interactions with co-factors, and serve as regulatory hubs in response to environmental signals (Zaharias et al., 2021; Bigman et al., 2022). These insights suggest that structural diversification in terminal regions of CsAP2/ERF proteins may underpin functional divergence and plasticity, reinforcing their roles in regulating complex developmental and adaptive processes in *C. sinense*.

### Gene duplication and cis-regulatory characteristics

Plants can rapidly adapt to environmental changes through gene family expansions driven by segmental and tandem duplications (Flagel and Wendel, 2009). In *C. sinense*, the distribution of tandem duplications was uneven, with 21 *CsAP2/ERF* genes clustered into 12 regions of tandem duplication across chromosomes 1, 4, 5, 6, 10, 12, 17, and 18. Additionally, 25 segmental duplication events involving 41 *CsAP2* genes were identified. These findings suggest that both tandem and segmental duplications have significantly contributed to the evolution of the *CsAP2/ERF* gene family.

Cis-acting elements in the promoters are crucial for transcriptional regulation, and polymorphisms in these regions often play an important role in gene expression variation (Rosas et al., 2014; Wang et al., 2021). Among the *CsAP2/ERF* genes, ABRE (abscisic acid response element) motifs were found widely distributed upstream of 88 genes, with 66 of these showing multiple occurrences. ABA treatment further confirmed the differential expression of these genes, highlighting their potential role in ABA-mediated stress responses.

### Expression profiling of CsAP2 genes and their potential regulatory networks

Orchids are one of the most diverse groups of angiosperms, with an estimated 25,000 - 30,000 species. *C. sinense*, a representative species of this family, is renowned for its extensive natural variation in flower organs, flower and leaf colors, and other traits. Confucius famously praised them as the “King of Fragrance” (Hew and Wong, 2023a). Floral patterning variation in orchids is primarily regulated by the MADS-box gene family (Li et al., 2022). Meanwhile, variations in leaf color, such as the yellowing observed in some varieties, result mainly from chlorophyll degradation and carotenoid accumulation (Gao et al., 2020; Cao et al., 2022). The red coloration in flowers and leaves is largely attributed to anthocyanin synthesis (Albert et al., 2010; Sunil and Shetty, 2022). Floral scent production is closely associated with terpene synthase (TPS) genes (Dötterl and Gershenzon, 2023). In this study, we identified several potential *CsAP2/ERF* downstream target genes that are involved in these key traits. These include 30 genes related to the MADS-box family, 26 genes involved in the anthocyanin biosynthesis pathway, 34 genes related to the photosynthetic system, and 8 genes related to terpene biosynthesis (TPS).

**Figure 7:**
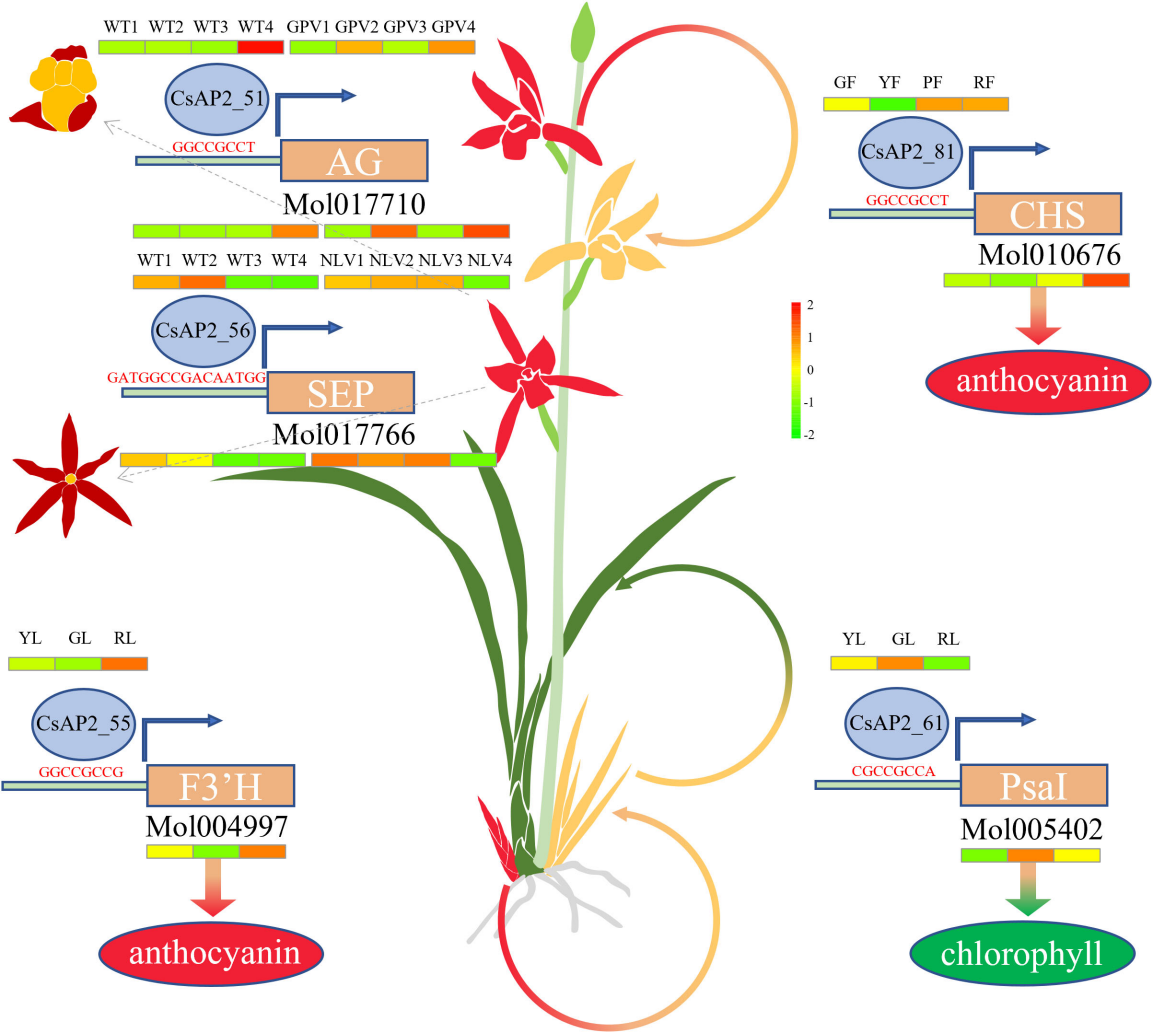
Model map of *CsAP2/ERF* gene regulation in the growth, development, and variation of *C. sinense*.

By integrating differential gene expression data with predicted target genes, we have mapped out a regulatory network that underlies key phenotypic traits in *C. sinense* (**Figure 7**). For example, *CsAP2_55* and *CsAP2_81* are implicated in regulating leaf and flower color. Specifically, *CsAP2_55* regulates leaf color by modulating the expression of the *F3’H* gene, which is involved in flavonoid biosynthesis, while *CsAP2_81* affects flower color by regulating the *CHS* gene, which plays a central role in the production of anthocyanins. Additionally, *CsAP2_61* indirectly regulates chlorophyll synthesis in leaves by influencing the expression of PsaI, a component of the photosystem I complex. A hallmark feature of orchids is the fusion of male and female reproductive organs into a gynostemium (Endress, 2015). In *C. sinense*, the *AGAMOUS* (*CsAG*) gene has been shown to play a critical role in gynostemium development (Su et al., 2018).

Our study finds that *CsAP2_51* binds to the promoter region of the *CsAG* gene and indirectly regulates gynostemium development. Furthermore, *CsAP2_56* may influence flower morphology by binding to the promoter of the *CsSEP* gene, potentially contributing to the formation of six-petaled flowers. These findings are further supported by predictions from AlphaFold3 (**Supplementary Figure 6**). These findings provide valuable insights into the functional roles of *AP2/ERF* genes in regulating key biological processes in *C. sinense*. The regulatory mechanisms we uncovered not only shed light on the molecular basis of traits such as flower and leaf color, floral scent, and gynostemium development, but also offer exciting directions for further research into the roles of these genes in other orchid species and angiosperms more broadly.

Through an integrative approach combining transcriptomic profiling, RT-qPCR validation, and detailed functional annotations, our study elucidates the extensive involvement of the CsAP2 gene family in both vegetative and reproductive developmental processes of *C. sinense*. Importantly, the yeast one-hybrid assay provided direct experimental evidence confirming that *CsAP2_51* binds specifically to the promoter region of *CsAG*, a key floral developmental regulator. This interaction points to a previously uncharacterized regulatory pathway potentially critical for floral morphogenesis and developmental plasticity in orchids. Collectively, these findings enhance our understanding of the evolutionary diversification and functional specialization within the AP2 transcription factor family. The regulatory insights and candidate genes described herein constitute an essential resource for future molecular and genetic studies aiming to unravel complex regulatory networks underlying orchid developmental biology and phenotypic diversification.

## Data Availability

Genome dataset and expression data are available at the genbank with accession number SAMN18586254.
