# Supplementary material for: Genome-wide analysis of AP2/ERF transcription Factors in Cymbidium sinense reveals their impact on orchid diversity
Source: Front Plant Sci. 2025 Jun 3;16:1541308. doi: 10.3389/fpls.2025.1541308 (PMC12170588; doi:10.3389/fpls.2025.1541308)
Supplement: Supplementary file 1 [file Presentation1.ppt]

## Slide 1
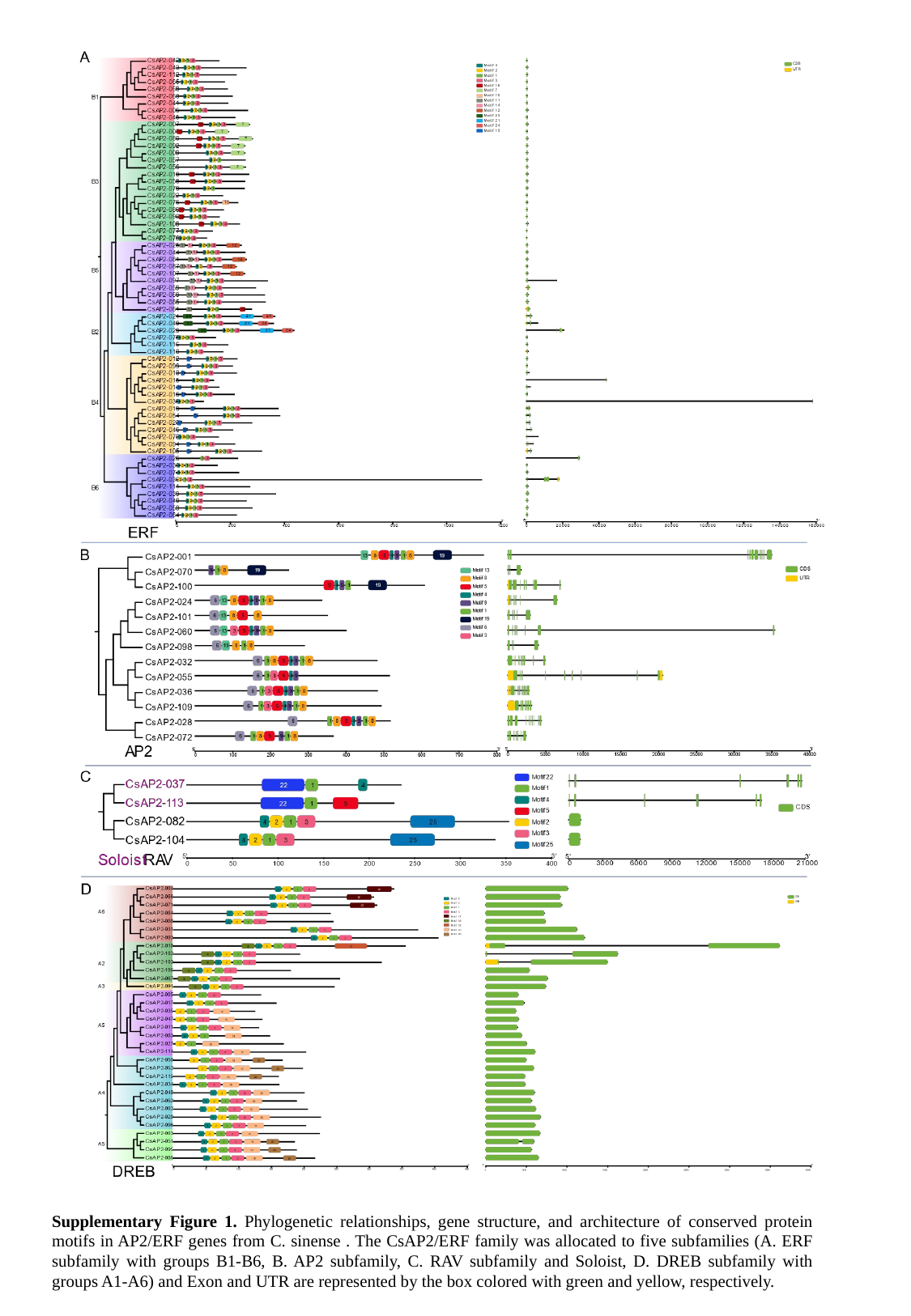

Supplementary Figure 1. Phylogenetic relationships, gene structure, and architecture of conserved protein motifs in AP2/ERF genes from C. sinense . The CsAP2/ERF family was allocated to five subfamilies (A. ERF subfamily with groups B1-B6, B. AP2 subfamily, C. RAV subfamily and Soloist, D. DREB subfamily with groups A1-A6) and Exon and UTR are represented by the box colored with green and yellow, respectively.

## Slide 2
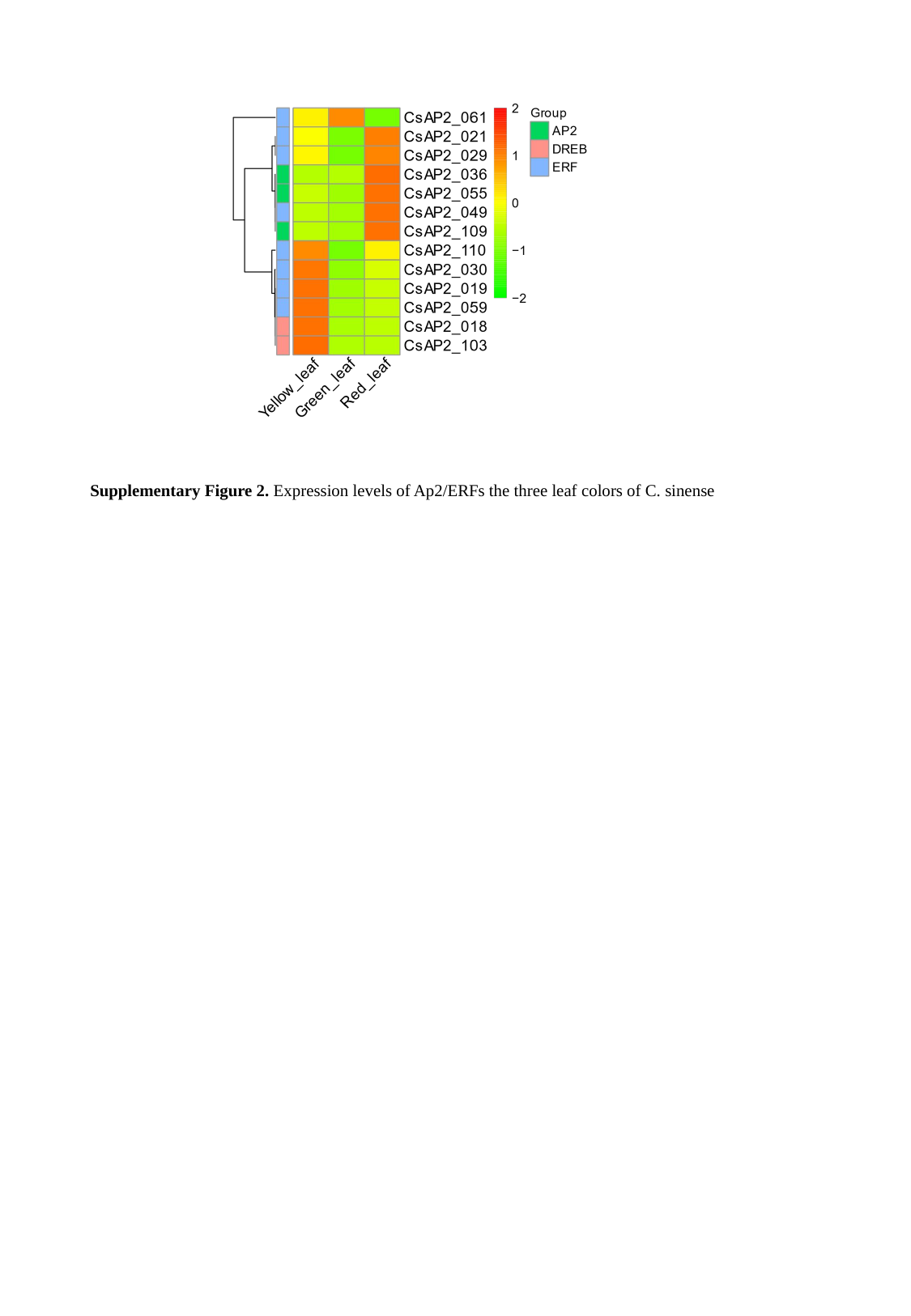

Supplementary Figure 2. Expression levels of Ap2/ERFs the three leaf colors of C. sinense

## Slide 3
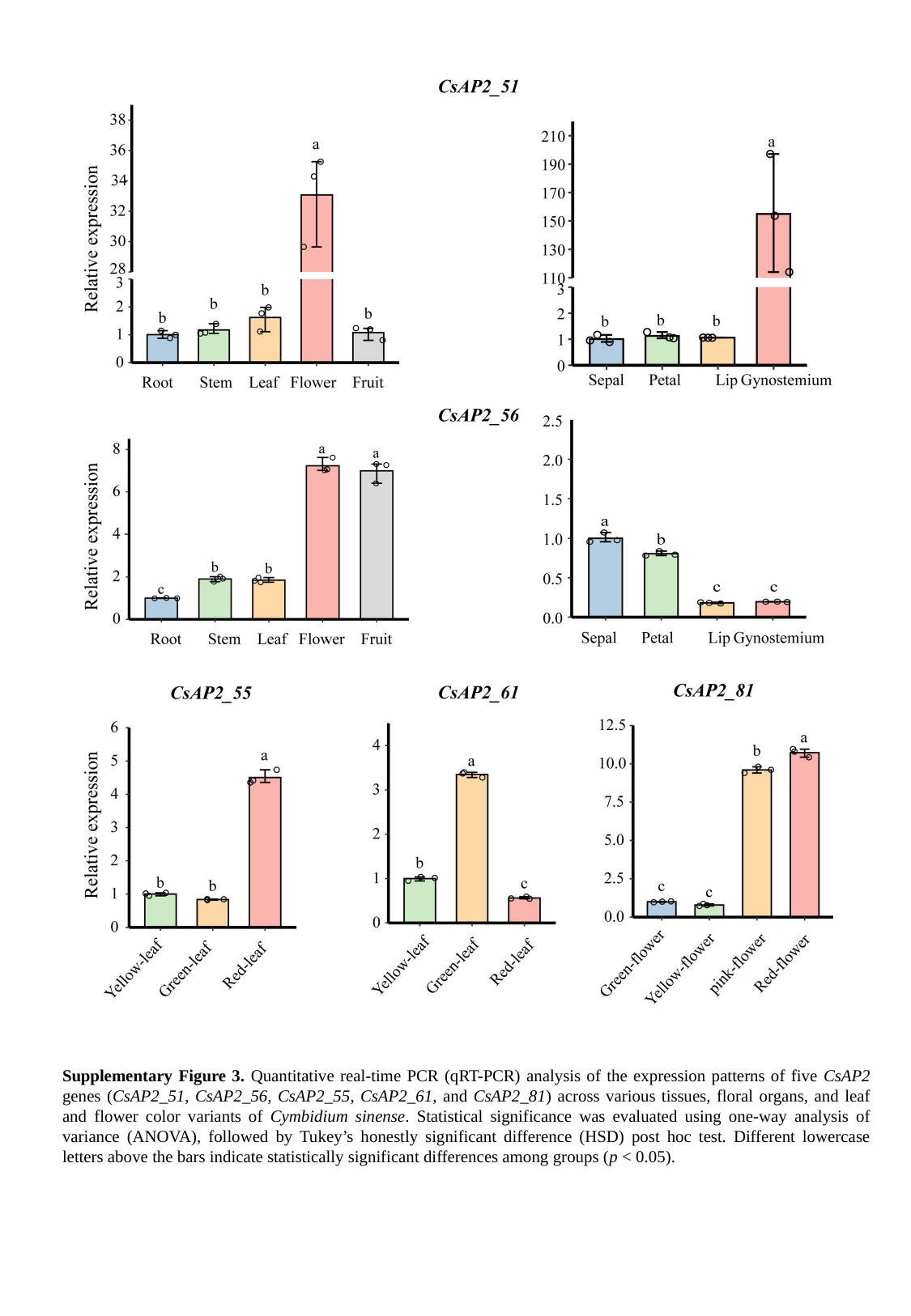

Supplementary Figure 3. Quantitative real-time PCR (qRT-PCR) analysis of the expression patterns of five CsAP2 genes (CsAP2_51, CsAP2_56, CsAP2_55, CsAP2_61, and CsAP2_81) across various tissues, floral organs, and leaf and flower color variants of Cymbidium sinense. Statistical significance was evaluated using one-way analysis of variance (ANOVA), followed by Tukey’s honestly significant difference (HSD) post hoc test. Different lowercase letters above the bars indicate statistically significant differences among groups (p < 0.05).

## Slide 4
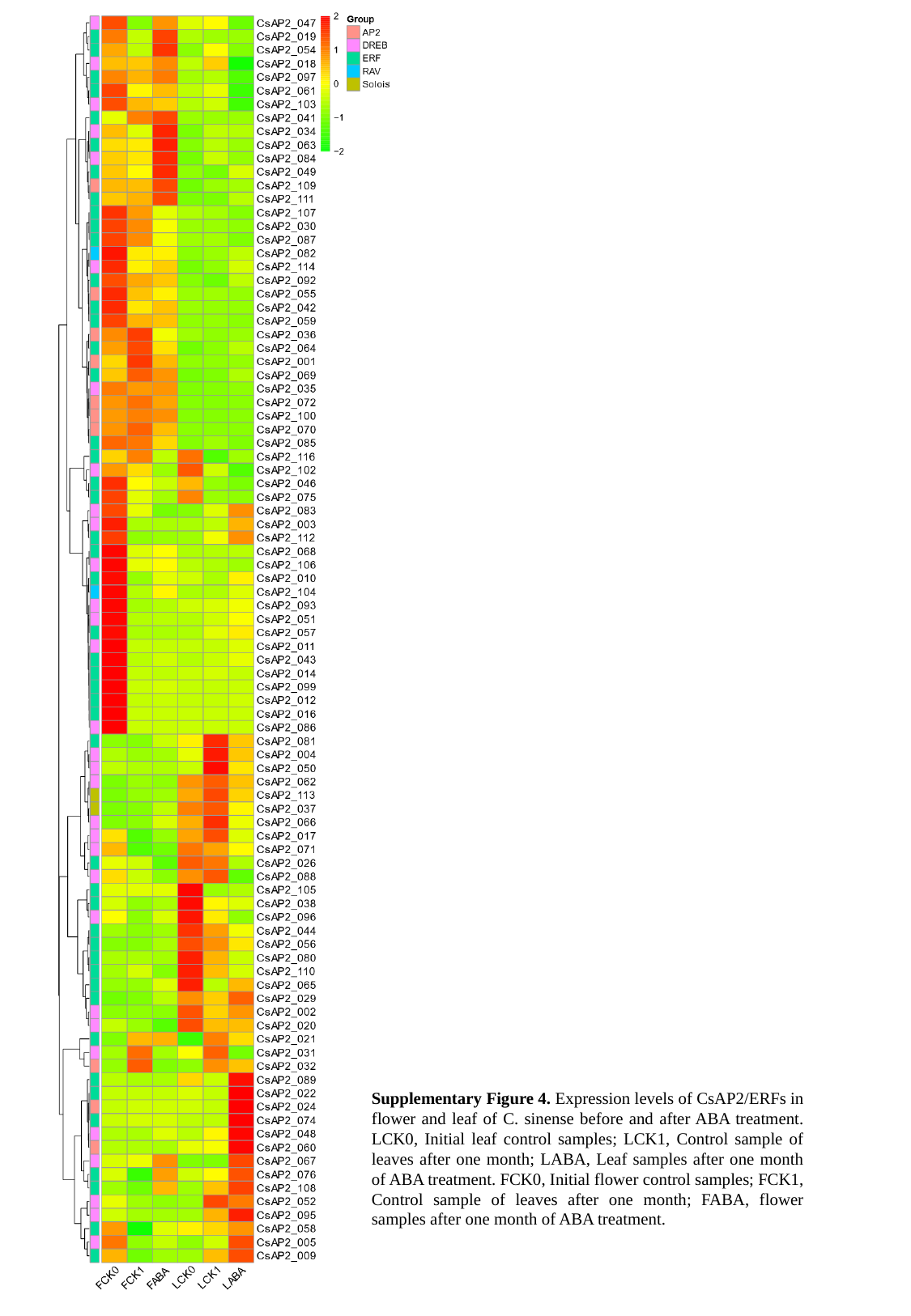

Supplementary Figure 4. Expression levels of CsAP2/ERFs in flower and leaf of C. sinense before and after ABA treatment. LCK0, Initial leaf control samples; LCK1, Control sample of leaves after one month; LABA, Leaf samples after one month of ABA treatment. FCK0, Initial flower control samples; FCK1, Control sample of leaves after one month; FABA, flower samples after one month of ABA treatment.

## Slide 5
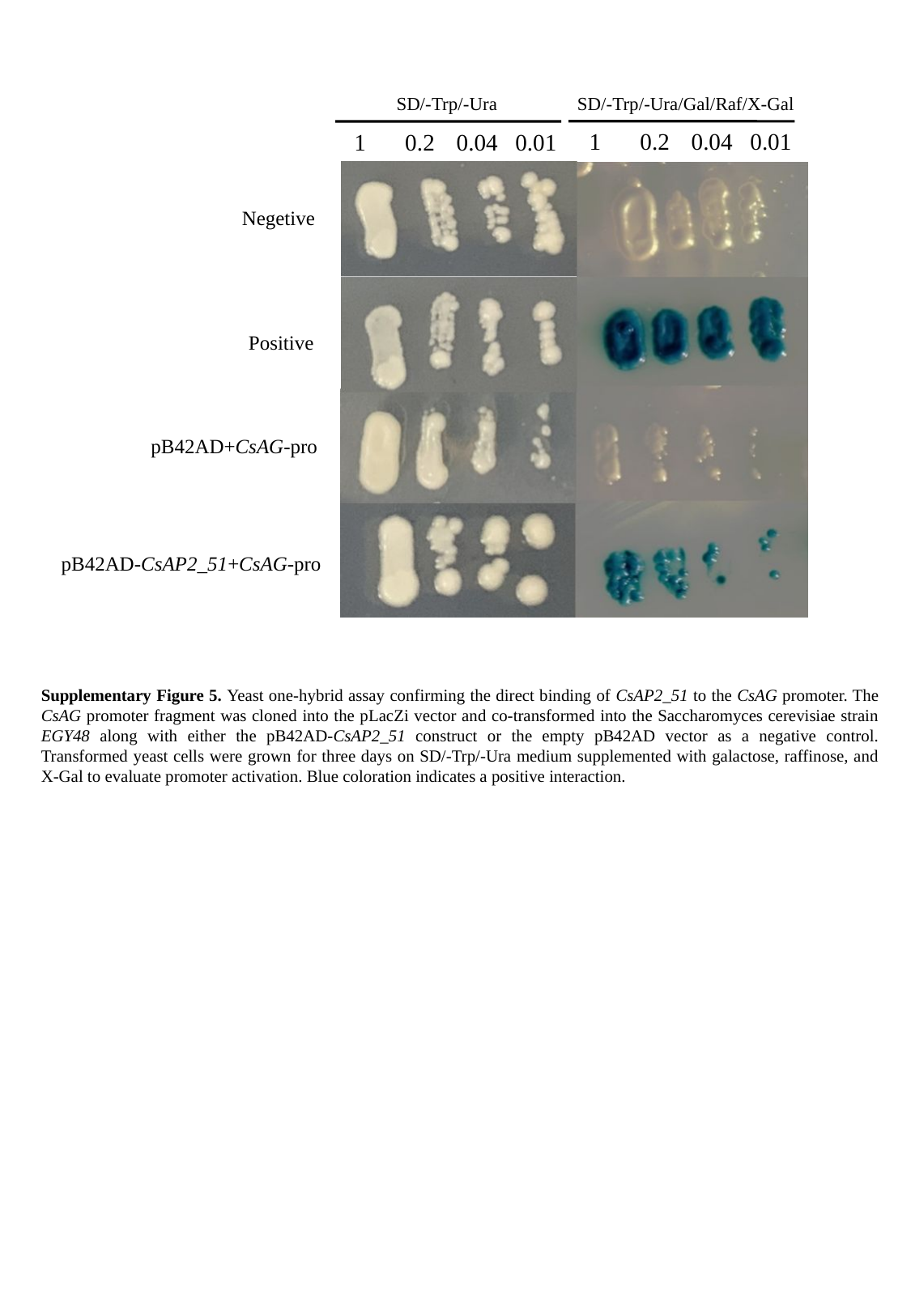

SD/-Trp/-Ura
SD/-Trp/-Ura/Gal/Raf/X-Gal
1
0.2
0.04
0.01
1
0.2
0.04
0.01
Negetive
Positive
pB42AD+CsAG-pro
pB42AD-CsAP2_51+CsAG-pro
Supplementary Figure 5. Yeast one-hybrid assay confirming the direct binding of CsAP2_51 to the CsAG promoter. The CsAG promoter fragment was cloned into the pLacZi vector and co-transformed into the Saccharomyces cerevisiae strain EGY48 along with either the pB42AD-CsAP2_51 construct or the empty pB42AD vector as a negative control. Transformed yeast cells were grown for three days on SD/-Trp/-Ura medium supplemented with galactose, raffinose, and X-Gal to evaluate promoter activation. Blue coloration indicates a positive interaction.

## Slide 6
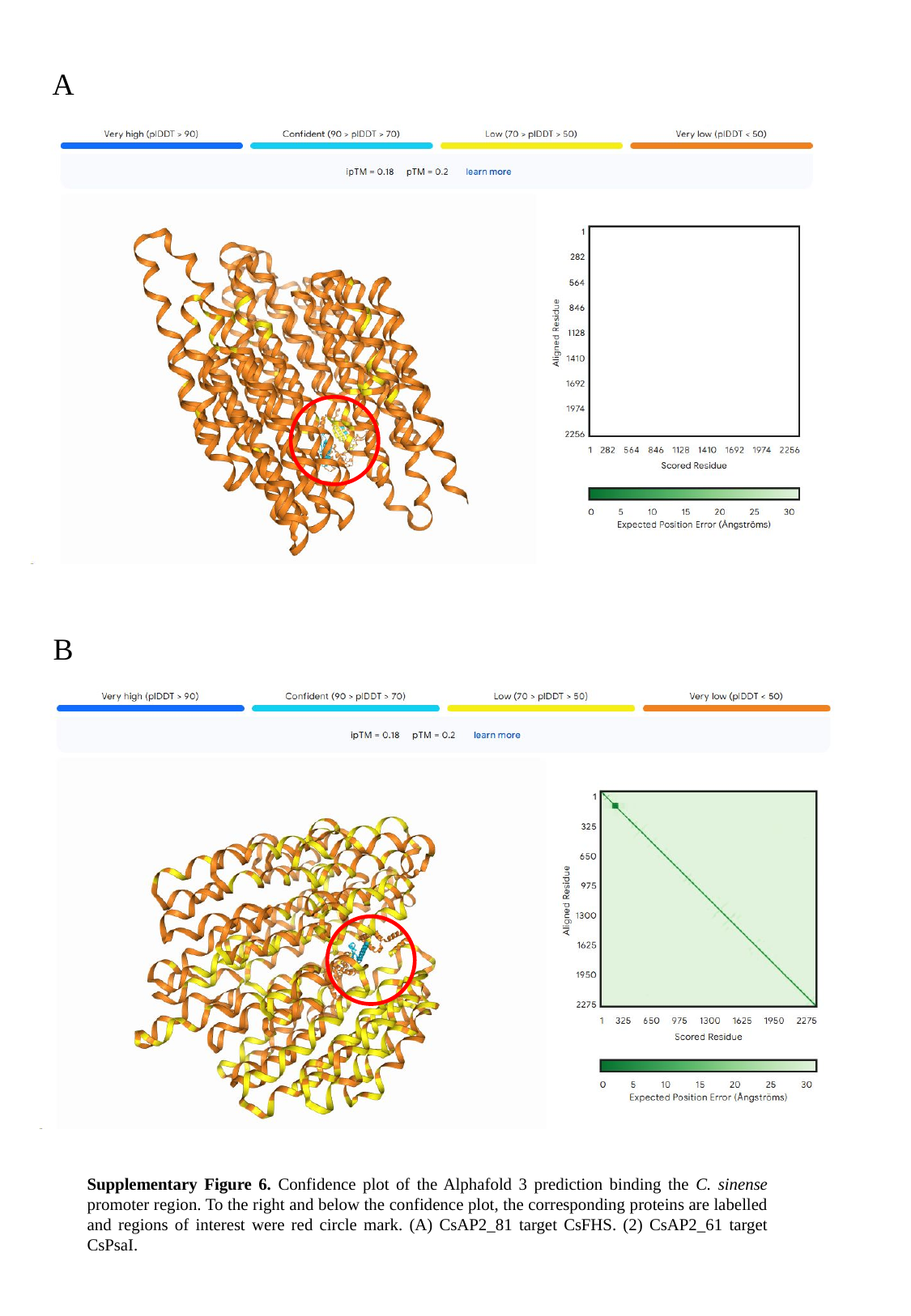

A
B
Supplementary Figure 6. Confidence plot of the Alphafold 3 prediction binding the C. sinense promoter region. To the right and below the confidence plot, the corresponding proteins are labelled and regions of interest were red circle mark. (A) CsAP2_81 target CsFHS. (2) CsAP2_61 target CsPsaI.
